# Characterization of Natural Killer Cells and Cytokines in Maternal Placenta and Fetus of Diabetic Mothers

**DOI:** 10.1155/2016/7154524

**Published:** 2016-05-16

**Authors:** Cristiane de Castro Pernet Hara, Eduardo Luzía França, Danny Laura Gomes Fagundes, Adriele Ataides de Queiroz, Marilza Vieira Cunha Rudge, Adenilda Cristina Honorio-França, Iracema de Mattos Paranhos Calderon

**Affiliations:** ^1^Graduate Program in Gynecology, Obstetrics and Mastology, Botucatu Medical School, São Paulo State University (UNESP), 18600-000 São Paulo, SP, Brazil; ^2^Institute of Biological and Health Science, Federal University of Mato Grosso, 78600-000 Barra do Garças, MT, Brazil

## Abstract

The present study characterized natural killer cells and cytokines in diabetic mothers, their placenta, and fetus. In the maternal blood from the hyperglycemic groups, the CD16^+^CD56^−^ NK cells increased, whereas that of CD16^+^CD56^+^ decreased in gestational diabetes mellitus [GDM] group. Cord blood from type 2 diabetes [DM-2] showed a higher proportion of CD16^+^CD56^−^ and CD16^−^CD56^+^. The placental extravillous layer of GDM and DM-2 showed an increase of CD16^+^CD56^−^ cells and, irrespective of region, the proportion of CD16^−^CD56^+^ cells was higher in mild gestational hyperglycemia [MGH] and GDM and lower in DM-2. IL-2 was lower in maternal blood and IFN-*γ* higher in maternal and cord blood from the GDM group. IL-17 was higher in maternal and cord blood from the DM-2 group. The placental extravillous layer of the MGH showed high levels of IL-4, IL-6, IL-10, IL-17, and IFN-*γ* and low levels of IL-1*β* and IL-8, whereas the placental villous layer contained high levels of IL-17 and IFN-*γ*. The GDM group, irrespective of region, showed higher levels of IL-8. The DM-2 group, irrespective of region, placenta showed high levels of TNF-*α*, IL-17, and IFN-*γ*. The hyperglycemia produces an inflammatory environment with a high content of inflammatory cytokines and cells expressing CD16^+^.

## 1. Introduction

During pregnancy, the maternal system undergoes adaptations involving cells and proteins associated with the immune-regulatory function to ensure fetal development. One of the tissues participating in immune-regulation of the maternal-fetal relationship is the placenta, a transient organ responsible for fetal protection, nutrition, breathing, and endocrine control [1]. Placental cells derived from both maternal and fetal organisms express molecules that play a fundamental role in maternal-fetal tolerance [2].

Decidua leukocytes, including macrophages, are a prominent population of T cells and natural killer (NK) cells [3] recognized by their direct* in vitro* cytotoxicity. NK cells are characterized as CD16 and CD56 according to the antigens on their surface. More than 95% of human uterine NK cells (uNK) do not exhibit cytotoxicity and are phenotypically defined as CD56 [4–6]. These cells can recognize the fetal HLA-G histocompatibility antigen and produce cytotoxicity suppressors [7, 8]. The balance between peripheral blood NK cells and regulatory NK cells (NKreg) during pregnancy is essential [9].

The profile of NK cells in the decidua is different from that of NK cells in peripheral blood. Other studies report that decidual NK cells produce cytokines such as IFN-*γ*, TNF-*α*, and IL-4 [9]. TNF-*α*, IL-1*β*, and IL-6 are essential in early pregnancy, but, with the evolution of gestation, high TNF-*α* levels can promote preeclampsia and gestational diabetes mellitus, while low IL-10 levels are associated with preterm birth [10–15]. Therefore, TNF-*α* production appears to be required for early pregnancy maintenance, whereas IL-10 plays a protective role in fetal development [16]. In diabetic mothers, IL-17 levels increase during pregnancy, and the cord blood of their newborns shows low IFN-*γ* levels [17].

In normal pregnancy, IL-4 produced by trophoblasts stimulates Th2 lymphocytes and increases the levels of inhibitory receptors in decidual NK cells, which maintain their inactive phenotype (CD16^−^CD56^+^) [7, 8]. The hyperglycemic condition, however, generates a proinflammatory environment capable of affecting fetal development, and the production of inflammatory cytokines can pose a risk to fetal health and promote the development of complications associated with diabetes in pregnancy [16].

The immune response associated with diabetes during pregnancy has yet to be completely understood, and the role of NK cells remains unknown. Diabetes possibly changes the expression of NK cells and cytokines in the maternal-placental-fetal unit. To investigate this hypothesis, the present study characterized the NK cells and cytokines of diabetic mothers, as well as their placentas and fetuses.

## 2. Materials and Methods

Placenta, maternal blood, and cord blood from diabetic mothers was evaluated in a cross-sectional study. The subjects attended the Diabetes and Pregnancy Facility, School of Medicine Obstetrics Course, UNESP, Botucatu, SP. This study was approved by the institutional Research Ethics Committee, and all the subjects gave informed written consent before entering the experimental protocol.

### 2.1. Subjects

Placenta and blood samples from pregnant women (18–45 years old) were analyzed by maternal glycemic status. Pregnant women with diabetes mellitus type 2 (DM-2) were referred to the Service with a confirmed diagnosis. Pregnant women underwent a 75 g oral glucose tolerance test [OGTT-75 g] [18] and glucose profile (GP) [19], which were applied in parallel between the 24th and 28th weeks of pregnancy. Altered GP were considered when any one value is found equal to or exceeding fasting glycemia of 90 mg/dL and postprandial level of 130 mg/dL [20]. The OGTT-75 g was altered when any of the following plasma glucose values are met or fasting glycemia of 92 mg/dL is exceeded: 1 h postload level of 180 mg/dL and 2 h postload level of 153 mg/dL [19]. According to the results of the OGTT-75 g and GP test, 55 pregnant women were classified into the following groups: nondiabetic [ND; normal 75 g OGTT and GP; N = 15], mild gestational hyperglycemia [MGH; normal 75 g OGTT and abnormal GP; N = 15], gestational diabetes mellitus [GDM; abnormal 75 g OGTT in pregnancy; N = 10], and diabetes mellitus type 2 [DM-2; abnormal 75 g OGTT prior to pregnancy; N = 15] [20].

The subjects continued attending the facility, irrespective of diagnosis, and the hyperglycemic patients followed a specific treatment for glycemic control [19].

### 2.2. Subject Follow-Up and Characterization

Patients with DM-2 or MGH were evaluated for GP with fasting and pre- and postprandial glycemic levels for 24 hours in two-week intervals until the 32nd week and then weekly until delivery. Glycemic control was assessed during pregnancy. Adequate glycemic control during pregnancy was defined as a glycemic mean of 120 mg/dL or less, and inadequate control was defined as a glycemic mean higher than 120 mg/dL. Thus, pregnant women with MGH were treated with a diet recommended by a dietitian and exercise; insulin therapy was applied when they had inadequate glycemic control. Patients with DM-2 were treated with a diet, exercise, and insulin therapy since the beginning of the pregnancy [20]. The ND pregnant women did not receive any type of intervention against hyperglycemia control.

### 2.3. Blood Sampling and Preparation of Blood Cells

Samples of 8 mL of maternal blood were collected in the morning at 36th week of pregnancy, prior to the beginning of labor. The umbilical cord blood was intradelivery-collected shortly after clamping. The maternal blood and umbilical cord blood were collected in Vacutainer tubes (Becton Dickinson, USA) treated with anticoagulant (EDTA). We centrifuged them at 160 G for 15 min to separate plasma from the cells. The plasma was stored at −80°C for later cytokines analysis. The isolation procedure was performed by centrifugation (40 min at 160 ×g), with Ficoll-Paque gradient (density 1.077 g/L; Sigma Chemical, St. Louis, USA). After centrifugation the supernatant was discarded, and the opaque bands at the interfaces between plasma and Ficoll-Paque-1077 containing cells were collected carefully by aspiration with a siliconized Pasteur pipet and transferred to tubes. NK cells were purified by positive selection with magnetic beads [21]. Cells were resuspended independently in serum-free medium 199 and used immediately for assays of flow cytometer.

### 2.4. Placenta Sampling and Preparation of Blood Cells

Placentas were collected at the moment of delivery and immediately washed with a saline solution. Samples of villous tissue were taken across the placenta halfway between the maternal and fetal sides and the large vessels were removed leaving only villous tissue [22, 23]. The basal plate was carefully dissected from the villous tissue and the amniochorionic membrane. Isolation of extravillous tissue was adapted from described procedure for amniochorionic cytotrophoblast isolation [23, 24]. These fragments were immediately stored in liquid nitrogen and later processed for cells separation. Placental fragments were macerated in PBS with Tween 20 supplemented with protease inhibitors (0.1 mM of phenylmethylsulfonyl fluoride; 0.1 mM of benzethonium chloride, 10 mM of EDTA, 20 UI of aprotinin, and 0.5% of BSA) in a proportion of 100 mg of tissue/mL, using a homogenizer Power Gen 125 (Fisher Scientific@). The homogenate was filtered and reserved from cytokines quantification and the sediment (cells) was fractioned by centrifugation (160 ×g, 40 min) through gradient density. The isolation procedure was performed by Ficoll-Paque (density 1.077 g/L; Sigma Chemical, St. Louis, USA). After centrifugation the supernatant was discarded, and the opaque bands at the interfaces between PBS and Ficoll-Paque-1077 containing cells were collected carefully by aspiration with a siliconized Pasteur pipet and transferred to tubes. NK cells were purified by positive selection with magnetic beads [21]. The cells were washed twice with 199 medium culture (Sigma Chemical, St. Louis, USA). The cells were used immediately for assays of flow cytometer.

### 2.5. Cell Subsets

To verify the presence of subsets of NK cells expressing CD16^+^ and/or CD56^+^ placenta and blood cells were stained with 5 *μ*L of anti-CD3^+^ PERCP, anti-CD16 FITC, and anti-CD56 PE, for 30 min at room temperature. Cells were washed and resuspended in phosphate-buffered saline (PBS) containing bovine serum albumin (BSA; Sigma, ST Louis, USA; 5 mg/mL) for flow cytometry analyses. Isotype controls (IgG1-FITC, IgG1-PE both from BD Biosciences) were used. A minimum of 10.000 cells were gated by size (FSC) and granularity (SSC) with a flow cytometer (FACSCalibur, BD Biosciences, USA). Data were analyzed using the Flowjo 7.2.5 software.

### 2.6. Quantification of Cytokines

Plasma blood and placenta homogenate were collected and stored at −80°C prior to analyses. The samples were thawed and cytokines [IL-1*β*, IL-2, IL-4, IL-6, IL-8, IL-10, IL-12, IL-17, TNF-*α*, and IFN-*γ*] were measured by cytometric bead array [CBA, BD Biosciences, USA] according to the manufacturer procedures. A flow cytometer was used for these analyses [FACSCalibur, BD Biosciences, USA]. The data were analyzed using the software FCAP Array 1.0 [CBA, BD Biosciences, USA].

### 2.7. Statistical Analysis

Data were expressed as the mean ± standard deviation (SD). The statistically significant difference was evaluated using the analysis of variance (ANOVA) for CD16^+^ and CD56^+^ expression and cytokines. Statistical significance was considered for *p* < 0.05.

## 3. Results

Clinical data of the mothers and newborns are shown in Table 1. In the first trimester of pregnancy, DM-2 mothers exhibited the highest body mass index (BMI) and glycemic index (*p* < 0.05). Glycated hemoglobin (HbA1c) levels were higher in GDM and DM-2, and the placental index was higher in the MGH, GDM, and MGH groups. The highest proportion of LGA newborns was found in MGH (Table 1).

The highest content of CD16^+^CD56^−^ NK cells in maternal blood was detected in GDM and DM-2, followed by MGH (Table 2). In the cord blood, the amount of CD16^+^CD56^−^ cells was higher in DM-2, and in the placental villous layer it was similar among the groups. In the placental extravillous layer, GDM and DM-2 contained the highest levels of CD16^+^CD56^−^ cells (Table 2).

In general, CD16^−^CD56^+^ cells were similar in maternal blood among the groups. In the cord blood, their frequency was higher in GDM and DM-2. In the placenta, irrespective of the layer, the CD16^−^CD56^+^ cell proportion was higher in MGH and GDM and lower in the DM-2 group (Table 2). CD16^+^CD56^+^ cells were less frequent in maternal blood and cord blood from GDM. In the placenta villous tissue they were lower in DM-2, whereas in placenta extravillous tissue they were higher in MGH and DGM and lower in DM-2 (Table 2).

Cytokines (IL-1*β*, IL-2, IL-4, IL-6, IL-8, IL-10, IL-12, IL-17, TNF-*α*, and IFN-*γ*) were assessed in the placenta, maternal blood, and cord blood from the groups (Table 3). The levels of IL-1*β* in placenta extravillous tissue were lower in MGH and GDM. IL-2 levels were lower in the maternal blood from GDM and placenta villi from MGH, whereas IL-4 levels were higher in the cord blood from GDM and placental extravillous layer from MGH (Table 3).

In MGH and GDM, IL-6 concentrations were higher in the placental extravillous layer and lower in placenta villi, and, in DM-2, they were higher in placenta villi. In GDM IL-8 levels were higher in placenta irrespective of the layer, and in DM-2 they were lower in the placental extravillous layer. MGH and DM-2 showed lower IL-8 levels in the placental extravillous layer (Table 3). IL-10 levels were higher in the placental extravillous layer from MGH, GDM, and DM-2. IL-12p70 concentrations were higher in the placental extravillous layer from DM-2 (Table 3).

Irrespective of placental section, TNF-*α* levels were higher in DM-2 and lower in GDM. IFN-*γ* levels were higher in maternal and cord blood from GDM. Cytokine levels in the placenta were higher in MGH and DM-2. In placenta extravillous tissue IFN-*γ* levels were lower in GDM. In maternal and cord blood, the highest IL-17 levels were found in DM-2, and in the placenta they were higher in MGH and DM-2 (Table 3).

The placenta villous/extravillous cytokine ratio is shown in Table 4. For IL-1*β*, IL-2, and IL-17, it was similar among the groups; for IL-4, it was lower in MGH and DM-2, and for IL-6 it was lower in MGH and GDM. DM-2 exhibited higher villous/extravillous IL-8, TNF-*α*, and IFN-*γ* ratio and lower ratio for IL-12p70. For IL-8, the ratio was lower in GDM; for TNF-*α* it was lower in GDM and MGH; and for IL-10 it was lower in MGH (Table 4).

## 4. Discussion

Fetal and placental tissues require suitable environment, under homeostasis, whereas the maternal body is affected by factors related to metabolic alterations. The fetus receives passive immunity from the mother, which is crucial for newborn adaptation to the extrauterine environment because it provides protection during the first months of life [25–28]. Studies have showed that umbilical cord blood plasma contains soluble NKG2D ligands that alter the Nk function and act as a mechanism of fetal-maternal tolerance in human pregnancy [29]. In the present study we characterized the NK cell and cytokine profile in maternal and cord blood as well as the placenta, showing their differences as a function of hyperglycemic status.

NK cells have distinct phenotypes with different functions in discrete tissues; they mediate immune homeostasis in peripheral blood and secondary lymphoid tissues and regulate uterine vascularization [30]. The present study showed an increase in the subsets of cells expressing the CD16^+^ marker in mothers from DM-2 and GDM. This response was similar in fetuses from DM-2, but not in fetuses from GDM. An interesting result was that cells expressing CD16^+^ were frequent in the placental extravillous layer but not in the villous layer. In the peripheral blood, NK cells are typically the largest subset of perforin-expressing cytotoxic lymphocytes and constitute 5 to 15% of total lymphocytes [31]. However, human uterine NK cells (uNK) are phenotypically CD56^+^, which are not cytotoxic [4–6].

Other studies report that a large number of uNK cells are necessary to support successful embryo implantation. In general, the two major types of NK cells are CD56 (bright) and CD56 (dim) cells. Most CD56 (bright) cells are not found in peripheral circulation but reside almost exclusively in the secondary lymph tissue, and their main activity is the production of cytokines, which increases the number of inhibitory receptors in decidual NK cells, maintaining the nonactivated phenotype (CD16^−^CD56^+^) [7, 8].

In the present study, the subsets of cells expressing CD56^+^ in placenta were higher in MGH and GDM and less frequent in DM-2. The molecular mechanisms determining NK cell phenotype have yet to be elucidated [32]. NK cells in the human uterus (uNK) and decidua (dNK) have a unique functional profile. They participate in tissue remodeling and neoangiogenesis, regulating fetal trophoblast invasion, placental vascularization, and especially cytokine and chemokine production [8, 33]. Studies showed that women with GDM have abnormal NK cell function due expression of surface receptors and cytokine production [34], whereas in women with type I diabetes mellitus alterations occur in immunological balances during pregnancy with proinflammatory systemic environment and greatest impact on CD56 Nk cells [35]. In the present study, the increase in CD16^+^ and decrease in CD56^+^ cell expression in the placenta from DM-2 suggest intensification of the innate immune response in diabetic mothers, possibly modulated by the secretion of proinflammatory cytokines that stimulate the NK cells [36]. Both pro- and anti-inflammatory cytokines have the ability to affect the activity of NK cells [37].

The hyperglycemia status of the women under study affected the cytokine profile in maternal blood and placental tissue. In GDM and DM-2, the levels of inflammatory cytokines such as IFN-*γ* and IL-17 were higher in maternal and cord blood. Similarly, earlier studies showed that mild gestational diabetes and gestational diabetes affect IFN-*γ* in cord blood and IL-17 in maternal blood, suggesting that IL-17 is an important immunological indicator of inflammation during diabetes progression [17].

Major changes in cytokine levels were observed in the placenta and were associated with its layer (extravillous layer at the maternal interface and villous layer at the fetal interface). In fact, in DM-2 the maternal portion of placenta contained high levels of IL-12, IL-17, TNF-*α*, and IFN-*γ*, and the fetal interface had high levels of IL-6, TNF-*α*, IL-17, and IFN-*γ*, indicating that the fetal environment is supplied with inflammatory cytokines. This inflammatory profile was weaker in placentas from GDM and MGH. In MGH, placental levels of IL-1*β* and IL-8 were low but those of IL-6, IL-17, and IFN-*γ* were high. In placenta villi in GDM, despite the high IL-8 levels, the concentration of IL-10 was low. Low placental IL-10 levels have been associated with fetal loss, premature delivery, and preeclampsia, irrespective of the prevalence of maternal diabetes [38].

In a previous study it was found that placental TNF-*α* levels are higher in diabetic women, suggesting that the previous inflammatory and hyperglycemic environment stimulates placental production of TNF-*α* in a full-term pregnancy [15]. TNF-*α* production by immune cells in response to different inflammatory stimuli is enhanced by membrane rupture in complicated pregnancies [21, 39].

Cytokine IL-12 is reported to potentiate IFN-*γ* production by NK cells [40, 41], which is likely related to the higher number of cells expressing CD16^+^ in the placenta of diabetic mothers (DM-2). One remarkable result is that the villous/extravillous IFN-*γ* and TNF-*α* ratio was higher in DM-2 placenta. It should be considered that during pregnancy immunological adaptations occur, but the hyperglycemia can alter these adaptations and be responsible for the greater frequency of complications in pregnant women with diabetes [42].

This finding corroborates the hypothesis that the placenta is capable of modifying the concentrations of cells and cytokines from the maternal blood before transferring them to the fetus and that this activity is affected by the inflammatory environment produced by hyperglycemia. An appropriate pro- or anti-inflammatory cytokine balance in decidua is required to support fetal development and must therefore be preserved during antimicrobial responses to prevent inflammation-induced tissue damage and maintain a successful pregnancy [21].

## 5. Conclusion

In conclusion, the levels of cells expressing CD16^+^ and cytokines in maternal blood, cord blood, and placental tissue are modified in pregnancies complicated by diabetes. Diabetic mothers release a larger amount of cytokines, indicating their important role in regulating the immune response during pregnancy. Despite our findings, changes in the profile of NK cells and cytokines in the placenta of patients with different types of hyperglycemia deserve further attention, given their function over the course of pregnancy.

## Figures and Tables

**Table 1:** Clinical data on the pregnant women with different glycemic status (ND: nondiabetic, MGH: mild gestational hyperglycemia, GDM: gestational diabetes mellitus, and DM-2: type 2 diabetes mellitus) and their newborns.

Parameters	ND (*N* = 15)	MGH (*N* = 15)	GDM (*N* = 10)	DM-2 (*N* = 15)
*Mothers *				
Age (years)	26.35 ± 4.20	27.63 ± 6.93	29.55 ± 6.55	32.55 ± 5.94
Gestational age (weeks)	39.17 ± 0.471	38.55 ± 1.39	38.12 ± 0.39	37.23 ± 1.29
Weight gain	13.02 ± 7.25	9.45 ± 4.78	11.31 ± 3.75	10.31 ± 6.75
BMI-1	27.52 ± 6.17	28.54 ± 7.05	28.66 ± 4.60	31.66 ± 6.96^*∗*^
BMI-2	31.13 ± 7.67	31.88 ± 6.50	32.89 ± 6.33	34.09 ± 9.93
Glucose level (mg/dL)	81.96 ± 9.60	93.73 ± 7.20	104.20 ± 15.87	105.30 ± 15.87^*∗*^
HbA1c (%)	5.07 ± 0.46	5.12 ± 0.44	5.79 ± 0.89^*∗*^	5.72 ± 0.44^*∗*^
*Newborn*				
Glucose (mg/dL)	77.71 ± 10.92	73.83 ± 15.08	59.04 ± 13.01	63.12 ± 16.11
Bilirubin	1.71 ± 0.48	1.74 ± 0.59	1.76 ± 0.80	1.82 ± 1.16
Insulin	>2	6.77 ± 2.77	6.65 ± 2.07	7.39 ± 5.7
Ponderal index	0.028 ± 0.002	0.028 ± 0.002	0.028 ± 0.003	0.028 ± 0.002
Placental index	0.168 ± 0.037	0.183 ± 0.0451^*∗*^	0.177 ± 0.03^*∗*^	0.187 ± 0.03^*∗*^
Birth weight and gestational age (%)				
SGA	14.28	8.00	7.50	9.20
AGA	78.57	60.00	79.00	76.92
LGA	7.15	32.00	13.50	13.88

Note: HbA1c: glycated hemoglobin; BMI-1 and BMI-2: body mass index in the first and third trimester of pregnancy, respectively. Data correspond to the median of 55 mothers and newborns. The ponderal index of a newborn is the cube root of body weight × 100 divided by height in centimeters; the placental index is the ratio of placental weight to fetal weight. SGA: small for gestational age; AGA: adequate for gestational age, and LGA: large for gestational age.

^*∗*^Statistical difference between ND and the other groups.

**Table 2:** Percentage of cell phenotypes CD16^+^ and/or CD56^+^ in the placenta, maternal blood, and cord blood of groups without diabetes (ND) or with mild hyperglycemia (MGH), gestational diabetes mellitus (GDM), and diabetes mellitus (DM-2).

Cells (%)	Sample	ND (*N* = 15)	MGH (*N* = 15)	DGM (*N* = 10)	DM-2 (*N* = 15)
CD16^+^ CD56^−^	Maternal blood	12.85 ± 6.73	30.67 ± 2.50^*∗*^	84.40 ± 16.36^*∗*^	57.27 ± 5.68^*∗*^
	Cord blood	18.53 ± 3.11	23.71 ± 2.16	15.43 ± 8.59	41.94 ± 18.98^*∗*^
	Placenta villi	4.70 ± 2.71	4.70 ± 2.71	4.19 ± 3.01	4.76 ± 2.48
	Placenta extravilli	4.85 ± 3.22	3.45 ± 1.64	10.65 ± 2.84^*∗*#^	10.20 ± 6.91^*∗*#^

CD16^−^ CD56^+^	Maternal blood	1.36 ± 0.19	1.90 ± 0.15	1.45 ± 0.26	1.45 ± 0.26
	Cord blood	0.50 ± 0.10	0.38 ± 0.15	2.76 ± 0.18^*∗*^	2.16 ± 0.18^*∗*^
	Placenta villi	13.37 ± 5.56	66.74 ± 20.19^*∗*^	22.26 ± 16.11^*∗*^	5.11 ± 3.10^*∗*^
	Placenta extravilli	16.49 ± 7.67	27.56 ± 13.00^*∗*#^	30.17 ± 19.00^*∗*^	8.54 ± 2.15^*∗*^

CD16^+^ CD56^+^	Maternal blood	8.88 ± 3.34	6.69 ± 2.25	3.35 ± 1.02^*∗*^	6.70 ± 2.25
	Cord blood	4.66 ± 2.56	5.87 ± 1.42	3.32 ± 2.20^*∗*^	5.88 ± 1.41
	Placenta villi	3.13 ± 1.2	4.70 ± 1.92	4.20 ± 1.18	1.56 ± 0.55^*∗*^
	Placenta extravilli	5.00 ± 1.1	7.46 ± 1.31^*∗*^	6.76 ± 1.04^*∗*#^	3.68 ± 1.47^*∗*#^

Note: results are expressed as the mean of samples of maternal blood, cord blood, and placental tissue. ^*∗*^Statistical difference between ND and the other groups; ^#^statistical difference between cells in placenta villi  and placenta extravilli  within the same group.

**Table 3:** Cytokine levels (pg/mL) in placental tissue, maternal blood, and cord blood in groups without diabetes (ND) or with mild hyperglycemia (MGH), gestational diabetes mellitus (GDM), and diabetes mellitus (DM-2).

Cytokines	Samples	Concentration (pg/mL)			
		ND (*N* = 15)	MGH (*N* = 15)	GDM (*N* = 10)	DM-2 (*N* = 15)
IL-1 *β*	Maternal blood	16.43 ± 2.71	16.33 ± 1.07	16.01 ± 2.00	16.85 ± 1.64
	Cord blood	15.49 ± 2.12	15.84 ± 0.79	16.54 ± 1.80	14.96 ± 2.39
	Placenta villi	10.78 ± 4.05	8.24 ± 0.66	9.68 ± 1.24	8.24 ± 0.49
	Placenta extravilli	12.14 ± 4.29	8.23 ± 2.04^*∗*^	9.76 ± 2.27^*∗*^	10.86 ± 2.58

IL-2	Maternal blood	15.34 ± 4.86	15.37 ± 3.74	9.76 ± 0.48^*∗*^	14.00 ± 4.02
	Cord blood	12.77 ± 3.20	14.05 ± 3.76	12.41 ± 2.74	11.76 ± 3.61
	Placenta villi	10.42 ± 0.46	7.28 ± 3.11^*∗*^	9.34 ± 3.90	10.05 ± 3.78
	Placenta extravilli	11.10 ± 1.20	10.21 ± 2.21	9.48 ± 0.87	10.70 ± 3.54

IL-4	Maternal blood	7.06 ± 1.82	10.16 ± 2.87	8.60 ± 0.93	8.30 ± 2.16
	Cord blood	7.76 ± 1.97	12.80 ± 5.90	14.76 ± 6.58^*∗*^	9.06 ± 4.40
	Placenta villi	8.93 ± 0.70	9.93 ± 3.93	7.84 ± 3.32	8.35 ± 0.67
	Placenta extravilli	9.34 ± 0.74	28.81 ± 13.32^*∗*#^	8.42 ± 0.82	12.85 ± 5.05

IL-6	Maternal blood	16.32 ± 2.45	15.36 ± 1.70	16.34 ± 1.96	15.06 ± 0.95
	Cord blood	16.29 ± 2.37	16.39 ± 1.54	16.20 ± 2.00	14.27 ± 1.40
	Placenta villi	20.29 ± 13.68	7.93 ± 0.67^*∗*^	10.26 ± 2.83^*∗*^	47.45 ± 17.10^*∗*^
	Placenta extravilli	11.82 ± 4.35^#^	35.67 ± 25.47^*∗*#^	39.28 ± 19.07^*∗*#^	10.03 ± 2.47^#^

IL-8	Maternal blood	24.5 ± 4.39	24.61 ± 1.41	25.44 ± 2.95	24.23 ± 2.68
	Cord blood	26.95 ± 4.95	26.41 ± 3.38	28.44 ± 4.12	23.24 ± 4.99
	Placenta villi	28.77 ± 19.81	19.32 ± 5.27	35.57 ± 12.03^*∗*^	30.80 ± 14.33
	Placenta extravilli	37.59 ± 21.26^#^	12.93 ± 5.51^*∗*#^	42.73 ± 24.56^#^	22.47 ± 4.99^*∗*#^

IL-10	Maternal blood	14.75 ± 1.78	13.91 ± 1.06	14.21 ± 1.13	13.95 ± 0.53
	Cord blood	14.40 ± 0.69	15.33 ± 1.91	14.58 ± 0.81	13.38 ± 1.06
	Placenta villi	13.59 ± 5.49	10.42 ± 2.74	9.08 ± 3.74^*∗*^	13.89 ± 6.98
	Placenta extravilli	10.66 ± 4.95^#^	26.45 ± 13.16^*∗*#^	26.51 ± 8.70^*∗*#^	27.16 ± 5.45^*∗*#^

IL-12p70	Maternal blood	10.89 ± 2.37	11.78 ± 4.46	11.37 ± 2.07	9.59 ± 1.05
	Cord blood	10.51 ± 1.25	11.79 ± 1.36	10.19 ± 0.52	11.19 ± 0.90
	Placenta villi	5.39 ± 1.22	5.76 ± 1.04	8.59 ± 4.81	10.68 ± 4.94
	Placenta extravilli	4.74 ± 0.51	7.9 ± 1.09	10.06 ± 3.60	15.34 ± 0.59^*∗*#^

TNF	Maternal blood	13.78 ± 2.82	13.27 ± 1.73	13.72 ± 1.67	13.34 ± 1.20
	Cord blood	13.74 ± 0.91	14.28 ± 1.15	12.54 ± 0.80	11.76 ± 0.75
	Placenta villi	7.48 ± 1.17	12.77 ± 5.18	8.88 ± 1.91	28.69 ± 13.82^*∗*^
	Placenta extravilli	10.61 ± 3.06^#^	14.36 ± 5.27	9.11 ± 4.51	17.99 ± 6.34^*∗*#^

IFN-*γ*	Maternal blood	6.37 ± 0.99	6.40 ± 0.71	22.83 ± 4.47^*∗*^	6.80 ± 1.55
	Cord blood	6.47 ± 0.99	17.05 ± 0.17	37.15 ± 11.98^*∗*^	8.45 ± 3.88
	Placenta villi	6.63 ± 1.02	12.88 ± 5.64^*∗*^	7.27 ± 0.15	19.95 ± 4.37^*∗*^
	Placenta extravilli	7.48 ± 1.18	14.11 ± 7.95^*∗*^	4.21 ± 2.14^*∗*#^	16.59 ± 4.92^*∗*^

IL-17A	Maternal blood	6.98 ± 1.11	7.08 ± 2.93	8.90 ± 2.45	16.20 ± 4.39^*∗*^
	Cord blood	5.25 ± 1.17	5.35 ± 0.26	8.67 ± 1.76^*∗*^	15.46 ± 2.78^*∗*^
	Placenta villi	5.15 ± 2.40	16.86 ± 5.64^*∗*^	5.11 ± 1.23	17.49 ± 5.39^*∗*^
	Placenta extravilli	5.63 ± 0.91	14.03 ± 4.78^*∗*^	7.69 ± 5.14	14.42 ± 7.20^*∗*^

Note: results are expressed as the mean of samples of maternal blood, cord blood, and placental tissue. ^*∗*^Statistical difference between ND and the other groups; ^#^statistical difference between cells in placenta villi and placenta extravilli within the same group.

**Table 4:** Placental villous/extravillous cytokine ratio in groups without diabetes (ND) or with mild hyperglycemia (MGH), gestational diabetes mellitus (GDM), and diabetes mellitus (DM-2).

Placental cytokines	Villous layer/extravillous layer ratio			
	ND (*N* = 15)	MGH (*N* = 15)	GDM (*N* = 10)	DM-2 (*N* = 15)
IL-1*β*	0.68 ± 0.23	1.04 ± 0.21	1.05 ± 0.18	0.79 ± 0.15
IL-2	0.94 ± 0.07	0.86 ± 0.17	1.00 ± 0.28	1.02 ± 0.34
IL-4	2.13 ± 1.09	0.45 ± 0.29^*∗*^	1.27 ± 0.38	0.70 ± 0.23^*∗*^
IL-6	1.13 ± 0.42	0.68 ± 0. 15^*∗*^	0.45 ± 0.08^*∗*^	1.02 ± 0.22
IL-8	0.75 ± 0.41	0.92 ± 0.20	0.25 ± 0.08^*∗*^	3.60 ± 1.90^*∗*^
IL-10	1.33 ± 0.58	0.52 ± 0.21^*∗*^	0.90 ± 0.35	0.75 ± 0.44
IL-12	1.36 ± 0.75	1.41 ± 0.95	1.40 ± 1.14	0.80 ± 0.33^*∗*^
IL-17	1.11 ± 0.49	1.33 ± 0.56	0.98 ± 0.20	1.50 ± 0.64
TNF-*α*	0.84 ± 0.13	0.83 ± 0.27^*∗*^	0.36 ± 0.10^*∗*^	1.50 ± 0.64^*∗*^
IFN-*γ*	0.80 ± 0.09	1.23 ± 0.74	0.64 ± 0.20	1.25 ± 0.28^*∗*^

Note: results are expressed as the mean of samples of placental tissue. ^*∗*^Statistical difference between ND and the other groups.
